# Flying squirrels, hidden treasures

**DOI:** 10.7554/eLife.89823

**Published:** 2023-07-07

**Authors:** Pizza Ka Yee Chow

**Affiliations:** 1 School of Psychology at the University of Chester Chester United Kingdom

**Keywords:** squirrel, behavior, environmental adaption, mortise-tenon structure, caching, tropical rainforest, food hoarding, Other

## Abstract

In the rain forests of Hainan, China, two species of squirrel create grooves on the surface of smooth nuts so that they can wedge them in the forks between branches.

**Related research article** Xu H, Xia L, Spence JR, Lin M, Lu C, Li Y, Chen J, Luo T, Li Y, Fang S. 2023. Flying squirrels use a mortise-tenon structure to fix nuts on understory twigs. *eLife* **12**:e84967. doi: 10.7554/eLife.84967.

Just how we may stash away precious chocolate bars for future cravings, many animals hoard food to prepare for times of low supply (Vander Wall, 1990). Tree squirrels, for instance, are well known for employing this strategy. Some, like the American red and Douglas squirrels, are predominantly ‘larder hoarders’ who tend to store nuts and seeds in one dedicated spot, called a midden (Steele, 1998; Steele, 1999). Others, like the Eurasian red squirrels and Eastern grey squirrels, tend to be ‘scatter hoarders’ who cache their items one by one in tree cavities, between logs, or inside holes in the ground (Smith, 1968; Wauters and Casale, 1996). Now, in eLife, Suqin Fang and colleagues from institutes in China and Canada — including Han Xu and Lian Xia as joint first authors — report a surprising hoarding strategy employed by certain Chinese populations of flying squirrels (Xu et al., 2023).

Xu et al. accidentally discovered this caching behaviour when conducting fieldwork in Hainan Island, China. Amongst the shrubs and saplings of the Hainan Tropical Rainforest National Park, they began to notice that many nuts from *Cyclobalanopsis* trees were ‘stuck’ within the fork present between diverging twigs. The smooth, oval-shaped nuts showed a spiral groove around their middle section, as well as chew marks of more varied depths towards their bottom end.

Intrigued, the team conducted three systematic field searches and set trail cameras to record animal activity around the clock. After months of work, a few images and videos revealed that two species of flying squirrels, *Hylopetes phayreielectilis* and *Hylopetes alboniger* (commonly known as the Indochinese and the particolored flying squirrels), were responsible for making these grooves and affixing the nuts (Figure 1).

**Figure 1.**
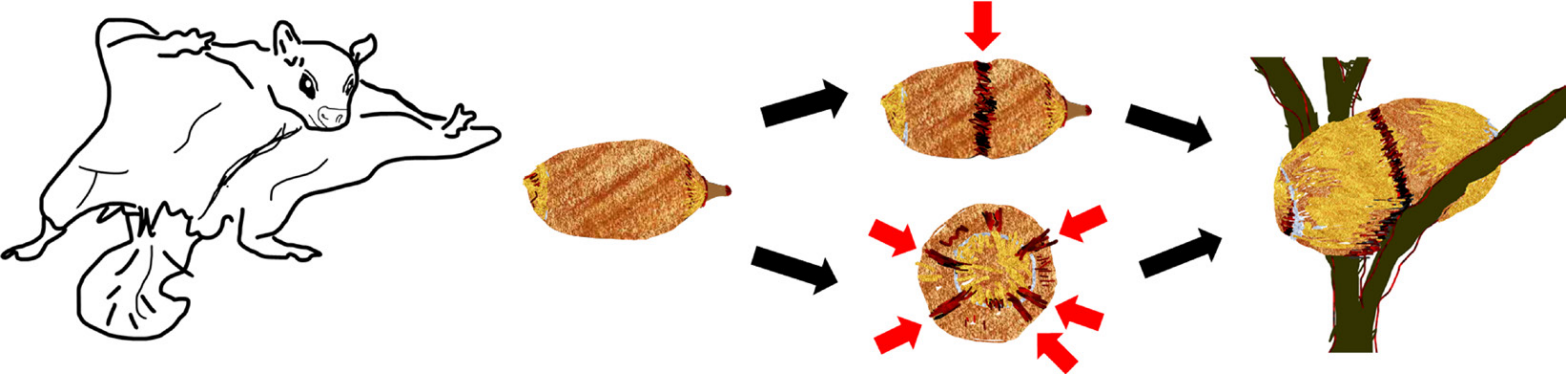
Food hoarding behaviour of two flying squirrel species, *Hylopetes phayreielectilis* and *Hylopetes alboniger* in Hainan Island, China. Upon finding a nut from the *Cyclobalanopsis* tree, two out of the nine species of flying squirrels present in Hainan rainforests make a spiral groove around the middle of the nut (top), as well as grooves at its bottom (bottom), before fixing it in the fork of twigs. This hoarding strategy may help to preserve the nuts for longer in this demanding, humid environment.

Importantly, this work suggests that the behaviour could represent an adaptive response to the demands of tropical forests. Tree squirrels face many challenges when it comes to hoarding their food, such as competitors that pilfer their stocks or environmental conditions that may degrade stored items. In response, they have developed a range of pre- and post-hoarding behaviours to minimise food loss (Robin and Jacobs, 2022). For example, to stop seeds and nuts from sprouting, American red squirrels store cones away from the soil in their middens while Eastern grey squirrels remove the structures responsible for germination from their acorns prior to hiding them (Steele et al., 2001; Vander Wall, 1990). Xu et al. noted that *Cyclobalanopsis* nuts would remain intact for several months once lodged within forks, whereas they germinate in two to three months when on the ground of tropical rainforests (Zhou, 2001).

Arguably, suspended nuts are there for all to see; this can result in thieving, a major social challenge that can lead hoarders to lose 30% of their food per day (Vander Wall, 1990; Vander Wall and Jenkins, 2003). The team recorded several types of behaviour that could help to minimize pilferage, including the animals often storing their nuts away from the tree where they had found them. Caches were also regularly visited by squirrels – a habit that could allow them to relocate items away from compromised locations, for example. Without knowing for certain that the same animal was both storing and checking on a particular nut, however, it is difficult to confirm that this indeed a post-hoarding strategy against thieves. Specifically identifying individuals in future field studies could help to better understand the factors that influence decision-making and behaviour before and after hoarding.

Many species of squirrel play a crucial role for forests, yet flying squirrels have often received less attention (Koprowski and Nandini, 2008). The findings by Xu et al. should renew interest in these rodents, as well as open new avenues of research in behavioural ecology and animal cognition. For example, tree squirrels are usually active during the day and recover their hoarded food using spatial memory alongside olfactory or visual cues; it is unclear if flying squirrels, which are active during the night, use these strategies as well (Gould, 2017; Li et al., 2018). It would also be interesting to investigate whether regularly visiting hoards serves as memory reinforcement to facilitate future recovery, as it is the case for other scatter-hoarding rodents such as agoutis (Hirsch et al., 2013). And finally, an exciting question remains unanswered: does grooving and affixing the nuts rely on trial-and-error and predisposed innate behaviours, or does it require planning and other advanced cognitive processes? More longitudinal observations and systemic experiments in the field will be needed to reveal the intricacies of this behaviour, and how it interacts with the demands of the environment.
